# Integrated metabolomics and electronic tongue analysis reveal dynamic changes in taste characteristics and non-volatile metabolites during Xinyang Maojian green tea processing

**DOI:** 10.1016/j.fochx.2026.104601

**Published:** 2026-10-14

**Authors:** Lanlan Peng, Lizeng Cheng, Kang Wei, Xinlin Wei

**Affiliations:** School of Agriculture and Biology, Shanghai Jiao Tong University, 800 Dongchuan Road, Shanghai 200240, PR China

**Keywords:** Xinyang Maojian, Green tea, Manufacture, E-tongue, Taste

## Abstract

As one of China's top ten famous teas, Xinyang Maojian (XYMJ) green tea lacks systematic research on dynamic variations of core taste metabolites throughout processing. This study combined metabolomics, multivariate statistics and electronic tongue evaluation to track its taste traits and metabolic fingerprints across sequential processing steps. 36 non-volatile metabolites were defined as pivotal differential metabolites. Fixation and initial drying were verified as decisive procedures shaping taste. Dynamic fluctuations of catechins, flavonoids, amino acids and carbohydrates jointly build sensory quality. Specifically, the improved in sweet taste attributed to the accumulation of soluble disaccharides (mainly sucrose), intensified umami was closely associated with the enrichment of glutamic acid, and the reduction in bitter and astringent aftertastes correlated with decreased levels of theobromine. Collectively, this study clarifies the key regulatory steps and target compounds during XYMJ green tea processing, providing theoretical basis for processing optimization and the directional improvement of its sensory quality.

## 1 Introduction

The distinctive flavor profile and health benefits of tea have made it increasingly popular among consumers, establishing it as the second most consumed beverage after water worldwide. Green tea is a type of non-fermented tea whose origins can be traced back to ancient China, as illustrated by the legend that “Shennong encountered seventy-two poisons in a single day while tasting hundreds of herbs, and was detoxified by tea” (Pan et al., 2022; Wu et al., 2020). The development of Western navigation later facilitated the introduction of Chinese green tea to Europe, where it gradually evolved into a globally popular drink. Over the past six decades, global green tea production and consumption have experienced sustained and significant growth, driven by the growing recognition of its health benefits (Ahmed & Stepp, 2024).

Regarded as one of China's top ten most renowned teas, Xinyang Maojian (XYMJ) stands out from numerous green tea varieties (Cui et al., 2022). The fresh tea leaves used to produce XYMJ are sourced from Xinyang, Henan Province, China. The local terrain consists of high mountains mostly ranging from 500 to 800 m above sea level, with rolling steep slopes shrouded in persistent clouds and mist all year round. The area experiences lower temperatures than many other tea-growing regions. These unique geographical and climatic conditions provide an exceptional natural environment for tea plant growth. Despite being hailed as the renowned teas, previous studies on XYMJ green tea have shown a disproportionate focus on the alterations in its aromatic profile during processing (Cui et al., 2022; Yin, Wang, et al., 2022). Limited attention has been focused on the effects of tea cultivar, growing altitude, and the fixation process on non-volatile metabolites (Wang, Cao, et al., 2021; Yin, Kong, et al., 2022). Consequently, a comprehensive understanding of the dynamic changes in key non-volatile metabolites throughout the entire manufacturing process and their direct impact on taste quality remains unclear.

The processing techniques of XYMJ green tea, including fixation, rolling, shaping, and drying, endow it with a distinctive flavor profile. Fixation rapidly deactivates enzymes in the fresh leaves, halting the enzymatic oxidation of polyphenols, thereby forming the “clear soup and green leaves” characteristic of green tea. It also evaporates some moisture, softening the leaves for subsequent rolling and shaping, while removing the grassy odor and developing the fragrance of the tea. Yin et al. reported that identified 14 key odorants as the primary aroma compounds in XYMJ green tea, while fixation process step contributed most significantly to the formation of its aroma profile (Yin, Wang, et al., 2022). After fixation, the leaves enter the rolling stage, where external force is applied to initially tighten and curl the strips. Rolling moderately ruptures the leaf cell structure, causing the tea juice to exude, which facilitates the infusion of internal substances during brewing. Cui et al. (2022) highlighted that rolling stage of XYMJ green tea promoted the disruption of leaf cells, which results in the release of seven kinds aroma-active compounds. The shaping process of XYMJ green tea is the core step in forming characteristic tight, thin, round, and straight appearance, while further dissipating moisture and setting. Drying is typically done in two stages: initial drying and re-drying, with a spreading-out period in between. The temperature for initial drying might be controlled between 70 and 90 °C, while re-drying uses a gentle, slow heat, aiming to gradually reduce the moisture content to about 5–6%. Drying thoroughly evaporates moisture, preventing the tea from spoiling and facilitating storage. It also further inhibits enzyme activity and enhances the aroma and taste of tea. These researches have demonstrated that the processing of XYMJ green tea directly influences the formation of its aroma profile. However, the dynamic changes of key taste-contributing substances during its manufacturing process remain unclear. Therefore, investigating the dynamic changes of metabolites during the processing of XYMJ green tea is essential to elucidate its key metabolic profile, thereby providing a theoretical basis for optimizing the quality of XYMJ green tea.

In recent years, metabolomics technology has been applied to systematically and comprehensively analyze the dynamic changes of metabolites during tea processing. Ultra-performance liquid chromatography-tandem mass spectrometry (UPLC-MS/MS) was employed to investigate the impact of key processing steps on green tea quality, revealing that the fixation process exerted the most profound influence on non-volatile metabolites, followed by drying and rolling processes (Wang, Hua, et al., 2021). Shan et al. (2024) utilized an integrated approach combining UPLC-MS/MS, electronic tongue (E-tongue) analysis to investigate the impact of the fixation process on the metabolic profile of Xihu Longjing green tea. The study revealed that increasing the fixation temperature promoted the content of cis-catechin compounds, while reducing the levels of epicatechin and dimeric catechins, thereby contributing to a reduction in the bitterness and astringency of the tea infusion. Nevertheless, conventional untargeted metabolomics faces considerable challenges in tea metabolite studies, particularly incomplete coverage of the metabolome and unsatisfactory identification accuracy. In this study, a tea mass spectrometry database which contains over 3000 standard compounds was employed to analyze metabolic changes during the XYMJ processing (Cheng et al., 2026). This approach can guarantee consistency in equipment and parameters required for tea metabolomics analysis, effectively reduced errors, and ensured data reproducibility. In addition, XYMJ green tea is highly esteemed for its distinctive sweetness. A targeted metabolomics analysis encompassing 32 sugars and their derivatives was employed to investigate the dynamic changes of sweetness-related compounds during the processing of XYMJ tea leaves.

This study aims to systematically investigate the dynamic changes of metabolites during the processing of XYMJ green tea. By utilizing a self-established untargeted metabolomics mass spectrometry database for tea, the research integrated metabolomics and electronic tongue analysis to characterize non-volatile compounds in XYMJ. Key processing steps that determine the taste quality of XYMJ green tea were elucidated. The findings provide a theoretical foundation for the refined processing of high-quality XYMJ green tea.

## 2 Materials and methods

### 2.1 XYMJ tea processing

The raw XYMJ tea samples of the seven different processing stages were provided by Guangyi Tea Co., Ltd. in Xinyang City, Henan Province, China. The XYMJ tea processing protocol incorporates an additional re-drying phase in the final drying stage, distinguishing it from conventional methods (Yin, Wang, et al., 2022). The full procedure is outlined below (Fig. 1A). (1) Fresh tea leaves (XY): The fresh tea leaves consisting of a single bud were harvested from a standard demonstration tea garden in Chenwan Village, Guangshan County, Xinyang City, Henan Province, China. All fresh leaf samples were randomly picked by tea farmers on April 1, 2024. (2) Fixing (SQ): The fresh tea leaves were spread at room temperature for 4 h and then subjected to a fixation process for 4 min at 200 °C using a 6CST-80 roller-type fixation machine (Shangyang, Zhejiang, China). (3) Rolling (RN): The fixed tea leaves were rolled for 30 min using a 6CR-40 rolling machine (Shangyang, Zhejiang, China). (4) Scattering (BT): The rolled tea leaves were scattered at 86 °C for 15 min. (5) Shaping (LT): After scattering, the shaping step was performed at 115 °C for 6 min using a 6CLZ-80/14 type strip-forming machine (Shangyang, Zhejiang, China). (6) Initial Drying (CH): Finally, the drying steps were performed using a 6CHB-10 type machine (Shangyang, Zhejiang, China), the tea leaves were initial dried at 80 °C for 20 min. (7) *Re*-drying (FH): After initial drying, the leaves were spread out for 30 min, and then the leaves were re-dried at 90 °C for 10 min.

**Fig. 1:**
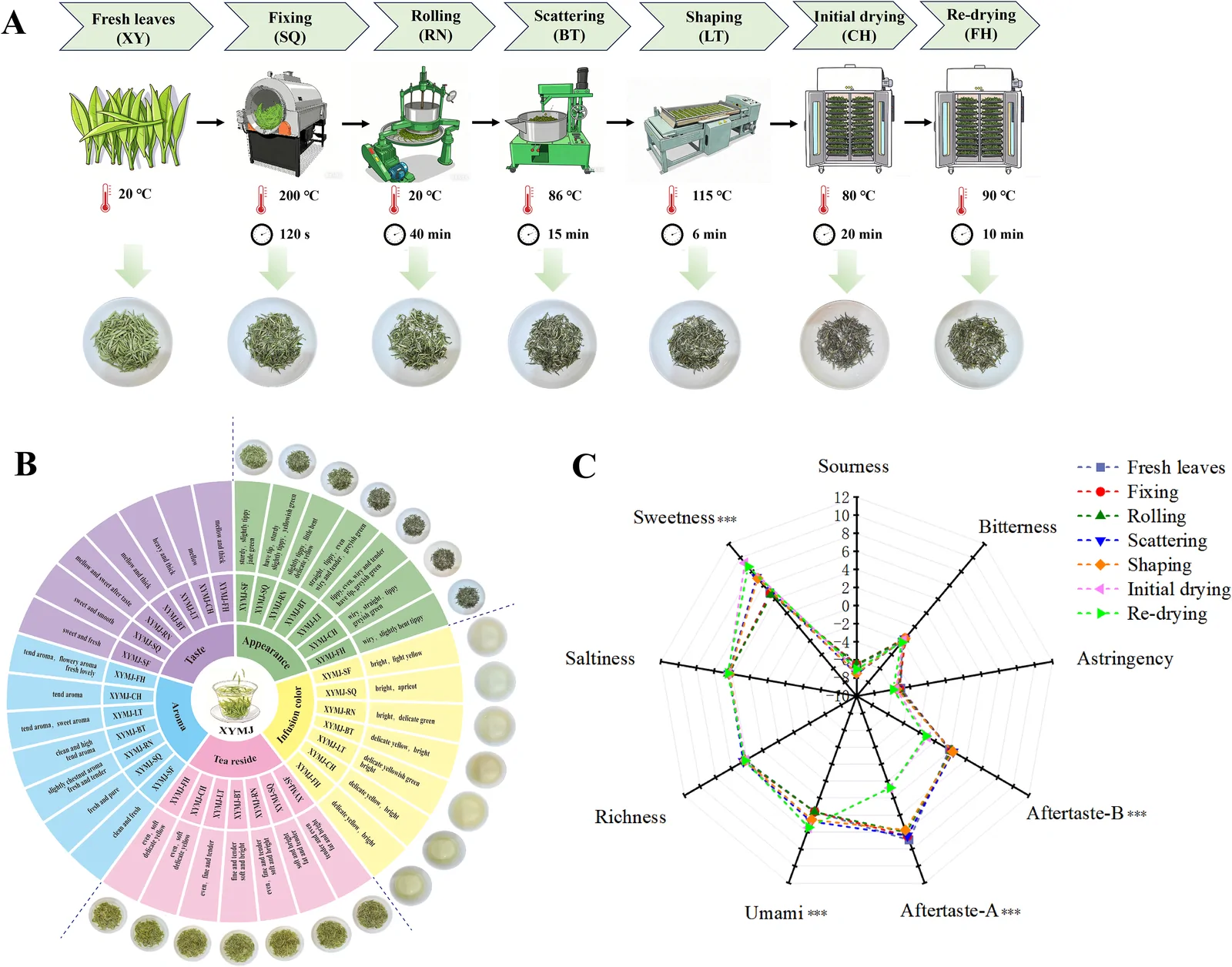
Dynamic changes in flavor during the processing of XYMJ tea. (A) The flow chart of XYMJ green tea manufacturing. (B) Flavor wheel analysis of tea samples from different processing steps of XMYJ tea. (C) Spider gram of taste quality of tea samples from different processing steps of XMYJ tea. *** represents significant differences (*p* < 0.001). (For interpretation of the references to color in this figure legend, the reader is referred to the web version of this article.)

### 2.2 Sensory evaluation

The sensory evaluation panel for XYMJ tea consisted of five panelists (3 males and 2 females) with extensive experience in tea sensory assessment. In accordance with the national standard GB/T 23776-2018, sensory evaluation was performed on the shape, liquor color, aroma, taste and infused leaves of XYMJ samples (Shan et al., 2024). Furthermore, an E-tongue taste analysis system was employed to dynamically track the taste quality of XYMJ tea samples at seven key processing stages (fresh leaves, fixation, rolling, scattering, shaping, initial drying, and re-drying). Briefly, precisely 3.00 g (dry weight) of crushed tea samples from each stage were weighed and extracted with 90 °C ultrapure water baths at a ratio of 1:30 (*w*/*v*) for 5 min. The extracts were cooled to room temperature and then filtered to obtain the tea infusion ready for testing. Taste analysis was performed using an E-tongue system (TS-5000Z, Insent, Japan). Utilizing an array of specific taste sensors, this system is designed to quantify key taste attributes: sourness, astringency, bitterness, saltiness, umami, sweetness, richness, bitter aftertaste, and astringent aftertaste.

### 2.3 Standard-verified untargeted metabolomic analysis

Sample preparation was performed according to the method in our previous study (Cheng et al., 2026). Briefly, tea samples were extracted three times with 70% methanol under ultrasonication. The supernatants were combined after centrifugation to form the analytical sample. Quality control (QC) samples were generated by pooling equal volumes of each sample extract prior to instrumental analysis.

Chromatographic separation was performed on a ACQUITY I-Class UPLC system (Waters, USA) equipped with a BEH C18 column (100 mm × 2.1 mm, 1.7 μm). A binary gradient elution was employed using 0.1% formic acid in water (mobile phase A) and 0.1% formic acid in acetonitrile (mobile phase B). The gradient program was set as follows: 0–5 min (5% B) → 5–12 min (20% B) → 12–17 min (100% B) → 18–20 min (5% B). The column temperature was maintained at 45 °C. The flow rate was 0.4 mL/min with an injection volume of 1 μL.

Mass spectrometry analysis was conducted using a quadrupole time-of-flight tandem mass spectrometer QTOF-MS/MS (Waters, USA) featuring an electrospray ionization (ESI) source, operating in positive ion mode. Key source parameters were configured as follows: drying gas temperature, 450 °C (flow rate 15 L/min); source temperature, 115 °C; capillary voltage, 2 kV; and cone voltage, 40 V. Full-scan mass spectra were acquired over the *m*/*z* range of 50–1500 with a resolution of 60,000 *fwhm*. For MS/MS analysis, data-independent acquisition (DIA) mode was applied using collision energies of 6 eV and a ramp of 20–45 eV. The mass axis was calibrated using leucine enkephalin (C₂₈H₃₇N₅O₇, [M-H]^−^ at *m/z* 554.2615). All UPLC-QTOF-MS/MS raw data were converted to mzML format and processed in MS-DIAL for peak picking, chromatographic alignment, peak matching, and normalization. The MS1 and MS2 mass tolerances for data acquisition were set to 0.01 Da and 0.025 Da, respectively. Peak detection was performed using a minimum peak amplitude of 1000 and a mass slice width of 0.1 Da, while chromatographic alignment was conducted with a 0.05 min retention time tolerance and 0.03 Da MS2 mass tolerance. Detected ion features were preliminarily annotated by matching accurate masses and fragmentation patterns against public databases (MSP, MassBank, ReSpect, Fiehn HILIC, GNPS) with 0.01 Da (MS1) and 0.05 Da (MS2) mass tolerances. Final compound confirmation was achieved via rigorous matching against the in-house Tea-MSDB (containing >3000 authentic standards and literature-reported tea metabolites), with thresholds of ±0.03 Da for accurate mass, ±0.05 min for retention time, and ± 0.10 Da for fragment ions.

### 2.4 Sugars analysis

Quantitative analysis of sugars and their derivatives in XYMJ tea samples was carried out via GC–MS. Briefly, freeze-dried and pulverized tea samples were mixed with 5 mL of 70% (v/v) methanol for ultrasonic-assisted extraction. Quality control (QC) samples were prepared by mixing equal proportions of all individual tea samples. GC–MS analysis was performed using a 7890B-7000D system (Agilent, CA) equipped with a DB-5MS capillary column (30 m × 0.25 mm × 0.25 μm). Chromatographic and mass spectrometric conditions were set according to previously established methods (Giordani et al., 2011; Kumar et al., 2022). A volume of 2 μL was injected in split mode (split ratio 3:1), with the column flow rate maintained at 1 mL/min. The oven temperature was initially held at 150 °C for 1 min, then raised to 200 °C at 5 °C/min, raised to 300 °C at 16 °C/min, and finally raised to 320 °C at 20 °C/min, followed by a 5.5 min hold. Quantitative analysis of monosaccharides, disaccharides, trisaccharides, and sugar derivatives in tea samples was conducted using external standard curves. Calibration curves were plotted with the concentration ratio as the abscissa and the peak area ratio as the ordinate. The selected ion monitoring mode was employed to eliminate matrix interference, thus ensuring higher sensitivity.

### 2.5 Statistical analysis

The data in this study are presented as mean ± standard deviation. A one-way Avalon method was used to test the significance of the data, with different letters indicating statistically significant differences (*p* < 0.05). OPLS-DA analysis of the untargeted metabolomics data was performed using Simca-P 14.1 (Umetric, Umea, Sweden). Heatmaps were generated via the Morpheus website (https://software.broadinstitute.org/morpheus/). Spearman's correlation analysis was used to assess the association between key differential metabolites and electronic tongue flavor profiles, and results were visualized on the GenesCloud platform (https://shiny.genescloud.cn/).

## 3 Results and discussion

### 3.1 Flavor wheel in the whole processing of XYMJ

As shown in Fig. 1B**,** the flavor wheel analysis comprehensively demonstrates the dynamic sensory variations of XYMJ green tea throughout its whole processing chain from five core sensory dimensions: tea appearance, liquor color, infused leaves, aroma and taste. Processing steps continuously reshape the morphological characteristics of dry tea, enrich endogenous aromatic substances, and enhance the fullness of tea liquor. The tea finished after re-drying delivers the best comprehensive sensory performance, characterized by uniform tender strips, bright tender-yellow liquor, fresh sweet honey aroma, and mellow, smooth taste with lasting sweet aftertaste. Fresh leaves retain their inherent fresh floral fragrance; fixation lays a foundational chestnut aroma. Rolling, strip straightening and shaping procedures mold the tea strips and intensify fresh aroma, whereas initial drying and re-drying fix the tea shape and upgrade its sweet and mellow flavor profile. The taste variations of XYMJ tea were further analyzed using an electronic tongue based on the taste sensing system (Fayaz et al., 2024; Steiner et al., 2024). The taste radar diagram revealed the taste quality changes across the XYMJ processing stages (Fig. 1C). As the processing steps progressed, the sweetness of XYMJ green tea gradually increased, while bitter aftertaste, and astringent aftertaste of XYMJ green tea decreased. Specifically, the scattering step and the initial drying step were crucial for enhancing the sweetness taste. The initial drying step was identified as the key process for reducing the bitter aftertaste and astringent aftertaste in quality formation of XYMJ green tea. Additionally, the drying process effectively enhanced the umami and sweet flavors of the tea leaves. Previous research has also confirmed the importance of drying in shaping the quality of green tea. For instance, Zhang et al. (2024) found that during the drying stage of Lu'an Guapian green tea, fire treatment significantly reduced bitterness and astringency while promoting the development of flavors such as floral, sweet, and roasted notes.

### 3.2 Overview of metabolites in the whole processing of XYMJ

The metabolic changes in XYMJ across seven distinct processing stages from fresh leaves to the finished product was performed. A total of 1335 metabolite ion features were detected form all samples. A total of 90 non-volatile metabolites were identified from seven distinct samples, including 27 flavonoids, 14 amino acids, 13 catechins and catechin derivatives, 7 organic acids, 6 lipids, 5 alkaloids, 4 nucleotides, 3 vitamins and cofactors, and 12 other compounds. The relative abundances of all metabolites are listed in Table S1. To elucidate the influence of different processing steps on tea metabolite profiles, Orthogonal Partial Least-Squares Discriminant Analysis (OPLS-DA) was employed to classify the samples from the seven processing stages (R^2^X = 0.767, R^2^Y = 0.609). The results are shown in Fig. 2A (PC1 = 0.325, PC2 = 0.0956). Obviously, samples from the seven different processing stages of XYMJ were separated into three distinct clusters, distributed in different quadrants. The unprocessed fresh tea leaves formed a separate group I. The samples from the fixing, rolling, scattering, and shaping stages clustered together as group II, exhibiting similar metabolite composition and abundance. Nevertheless, the initial drying and re-drying samples showed analogous metabolite profiles and served as group III. The Q2Y of supervised OPLS-DA model achieved 0.427. A permutation test with 200 iterations was conducted to evaluate overfitting, and the Q2 intercept was −0.431. The model revealed partial separation among sample groups without severe overfitting, whereas its predictive capacity was moderate.

**Fig. 2:**
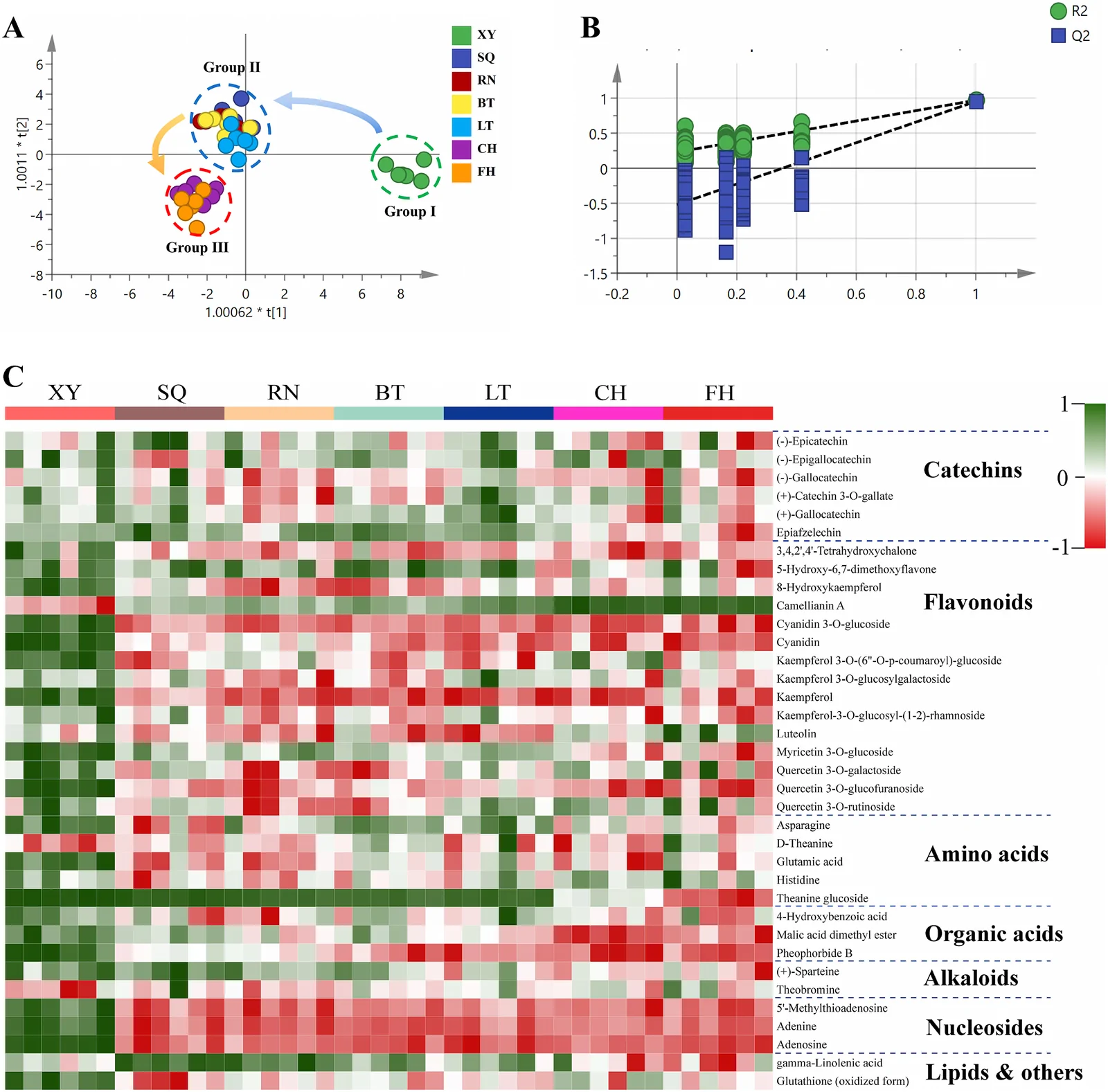
Multivariate statistical analysis of XYMJ tea samples at different processing stages. (A) OPLS-DA score scatter plot based on the composition of non-volatile metabolites. (B) Permutation test for validating the model. (C) Heatmap analysis of the 36 differential metabolites throughout the processing stages of XYMJ, and the relative abundance of metabolites is shown by a color gradient from red (high) to green (low). (For interpretation of the references to color in this figure legend, the reader is referred to the web version of this article.)

To elucidate the detailed phytochemical changes associated with key processing steps and taste quality differences in XYMJ tea, 36 key differential metabolites were identified, including 6 catechins and their derivatives, 15 flavonoids and their glycosides, 5 amino acids, 3 organic acids, 2 alkaloids, 3 nucleosides, 1 lipids and 1 other compounds. These metabolites were selected based on a variable importance in projection (VIP) > 1. The VIP values and MS/MS fragments are summarized in Table 1. A heatmap was generated to visually illustrate the dynamic changes in these key differential metabolites across different processing stages (Fig. 2C). Overall, the heat-map reveals that the most prominent metabolic changes in response to different processing stages are associated with flavonoids and their glycosides, catechin derivatives, and amino acids.

**Table 1:** The critical compounds (VIP > 1.00) responsible for the chemical variation.

NO.	Rt (min)	*m/z*	Formula	VIP	Metabolite name	XY	SQ	RN	BT	LT	CH	FH
1	4.48	291.07	C₁₅H₁₄O₆	1.09	(−)-Epicatechin	1.00abc	0.92c	0.99abc	1.02abc	0.93bc	1.08a	1.06ab
2	3.54	307.07	C₁₅H₁₄O₇	1.21	(−)-Epigallocatechin	1.00b	1.19a	1.06ab	1.03b	1.04b	1.05b	1.08ab
3	3.97	305.05	C₁₅H₁₄O₇	1.10	(−)-Gallocatechin	1.00b	1.04b	1.17a	1.10b	1.05b	1.18a	1.13b
4	3.75	443.08	C₂₂H₁₈O₁₀	1.25	(+)-Catechin 3-O-gallate	1.00b	1.10ab	1.26a	1.17a	0.96b	1.09ab	1.11ab
5	3.96	307.07	C₁₅H₁₄O₇	1.33	(+)-Gallocatechin	1.00bc	1.00bc	1.10a	1.00bc	0.96c	1.10a	1.05ab
6	5.06	275.08	C₁₅H₁₄O₅	1.17	Epiafzelechin	1.00b	0.95b	0.99b	0.93b	0.97b	1.15a	1.23a
7	6.50	273.06	C₁₅H₁₂O₅	1.19	3,4,2′,4’-Tetrahydroxychalone	1.00b	1.25a	1.29a	1.30a	1.26a	1.34a	1.29a
8	3.25	299.06	C₁₇H₁₄O₅	1.01	5-Hydroxy-6,7-dimethoxyflavone	1.00ab	1.01ab	1.04ab	0.94b	1.02ab	1.13a	1.16a
9	5.82	303.04	C₁₅H₁₀O₇	1.05	8-Hydroxykaempferol	1.00c	1.31b	1.57a	1.55a	1.35b	1.35b	1.30b
10	15.34	621.24	C₂₉H₃₂O₁₅	1.00	Camellianin A	1.00a	0.56b	0.52b	0.49b	0.40c	0.26d	0.27d
11	5.90	449.09	C₂₁H₂₁O₁₁^+^	1.02	Cyanidin 3-O-glucoside	1.00b	1.72a	1.84a	1.76a	1.81a	1.86a	1.80a
12	10.17	287.04	C₁₅H₁₁O₆^+^	1.11	Cyanidin	1.00c	1.96b	1.82b	2.17ab	2.35a	2.19ab	2.41a
13	9.13	595.12	C₃₀H₂₆O₁₃	1.21	Kaempferol 3-O-(6”-O-p-coumaroyl)-glucoside	1.00c	1.58a	1.33ab	1.53a	1.60a	1.18bc	1.44ab
14	3.75	611.12	C₂₇H₃₀O₁₆	1.24	Kaempferol 3-O-glucosylgalactoside	1.00bc	1.10abc	1.26a	1.21ab	0.99c	1.14abc	1.15abc
15	6.53	287.04	C₁₅H₁₀O₆	1.04	Kaempferol	1.00d	1.52c	1.68ab	1.68ab	1.71a	1.67ab	1.55bc
16	3.75	595.12	C₂₇H₃₀O₁₅	1.08	Kaempferol-3-O-glucosyl-(1–2)-rhamnoside	1.00c	1.15ab	1.26a	1.21ab	1.09bc	1.23ab	1.27a
17	3.75	287.04	C₁₅H₁₀O₆	1.13	Luteolin	1.00bc	1.08ab	1.13a	1.08ab	1.15a	0.99bc	0.90c
18	5.08	481.08	C₂₁H₂₀O₁₃	1.03	Myricetin 3-O-glucoside	1.00c	1.20b	1.15b	1.22b	1.17b	1.39a	1.42a
19	5.59	465.09	C₂₁H₂₀O₁₂	1.10	Quercetin 3-O-galactoside [Hyperoside]	1.00b	1.17b	1.35a	1.34a	1.14b	1.12b	1.13b
20	5.31	465.09	C₂₁H₂₀O₁₂	1.02	Quercetin 3-O-glucofuranoside	1.00b	1.67a	1.78a	1.64a	1.60a	1.83a	1.84a
21	5.59	611.14	C₂₇H₃₀O₁₆	1.19	Quercetin 3-O-rutinoside	1.00b	1.10b	1.32a	1.25a	1.01b	1.02b	1.05b
22	0.65	133.06	C₄H₈N₂O₃	1.38	Asparagine	1.00d	1.44a	1.22bc	1.06d	1.11 cd	1.29b	1.34ab
23	0.79	175.10	C₇H₁₄N₂O₃	1.02	D-Theanine	1.00a	0.94ab	0.94ab	0.87b	0.93ab	0.98a	0.88b
24	0.68	148.05	C₅H₉NO₄	1.07	Glutamic acid	1.00b	1.35a	1.35a	1.22a	1.24a	1.37a	1.25a
25	0.63	156.07	C₆H₉N₃O₂	1.11	Histidine	1.00b	1.22a	1.18a	1.15ab	1.09ab	1.02b	1.01b
26	1.18	337.15	C₁₃H₂₄N₂O₈	1.18	Theanine glucoside	1.00e	1.41de	1.91 cd	2.21c	1.89 cd	7.39b	13.57a
27	6.42	139.03	C₇H₆O₃	1.13	4-Hydroxybenzoic acid	1.00b	1.24a	1.26a	1.15ab	1.11ab	1.23a	1.26a
28	10.06	163.03	C_6_H_10_O_5_	1.03	Malic acid dimethyl ester	1.00e	1.14de	1.29 cd	1.18 cd	1.33bc	1.59a	1.48ab
29	15.88	607.26	C₃₅H₃₄N₄O₆	1.05	Pheophorbide B	1.00d	2.79c	2.90c	3.95b	3.99b	4.54a	4.20ab
30	13.49	235.16	C₁₅H₂₆N₂	1.10	(+)-Sparteine	1.00c	0.98c	1.09bc	1.12bc	1.28ab	1.37a	1.39a
31	3.70	181.07	C₇H₈N₄O₂	1.04	Theobromine	1.00a	0.92b	0.97ab	0.93ab	0.91b	0.93ab	0.90b
32	3.83	298.08	C₁₁H₁₅N₅O₃S	1.22	5′-Methylthioadenosine	1.00b	1.89a	1.93a	1.84a	1.78a	1.89a	1.89a
33	2.22	136.06	C₅H₅N₅	1.04	Adenine	1.00b	2.33a	2.34a	2.36a	2.28a	2.21a	2.28a
34	2.21	268.09	C₁₀H₁₃N₅O₄	1.06	Adenosine	1.00c	5.83b	5.47b	5.93b	6.16b	5.56b	6.13b
35	14.61	279.22	C₁₈H₃₀O₂	1.07	gamma-Linolenic acid	1.00bc	0.42d	0.49d	0.74 cd	0.78 cd	1.19ab	1.42a
36	1.59	613.13	C₂₀H₃₂N₆O₁₂S₂	1.25	Glutathione (oxidized form)	1.00c	1.75a	1.49b	1.50b	1.53b	1.35b	1.48b

Different letters a,b…… indicates statistically significant differences (*p* < 0.05).

Compared with Group I, Group II showed up-regulation of 37 metabolites and down-regulation of 3 metabolites (Fig. 3A). Compared with Group II, Group III exhibited up-regulation of 7 metabolites and down-regulation of 1 metabolite (Fig. 3B). Compared with Group I, 45 metabolites were up-regulated and 1 metabolite was down-regulated in Group III (Fig. 3C). Furthermore, 37 differential metabolites were identified as common to both the Group I vs Group III and Group I vs Group II comparisons (Fig. 3D). These results indicate that the processing steps beginning with fixation significantly altered the metabolic profile of the tea leaves. As shown in Table 1, among these differential metabolites, a total of 20 non-volatile compounds showed a sharply increase after SQ processing, primarily including cyanidin, pheophorbide B, 5′-methylthioadenosine, adenine, and adenosine. Additionally, 8 non-volatile compounds were markedly elevated after initial drying processing, mainly comprising theanine glucoside, γ-linolenic acid, and myricetin 3-O-glucoside, while 3 non-volatile compounds decreased significantly at this stage, including camellianin A, kaempferol 3-O-(6″-O-p-coumaroyl)-glucoside, and luteolin. Precisely identifying the differential metabolites associated with key processing steps provides a scientific basis for optimizing the manufacturing process of XYMJ green tea.

**Fig. 3:**
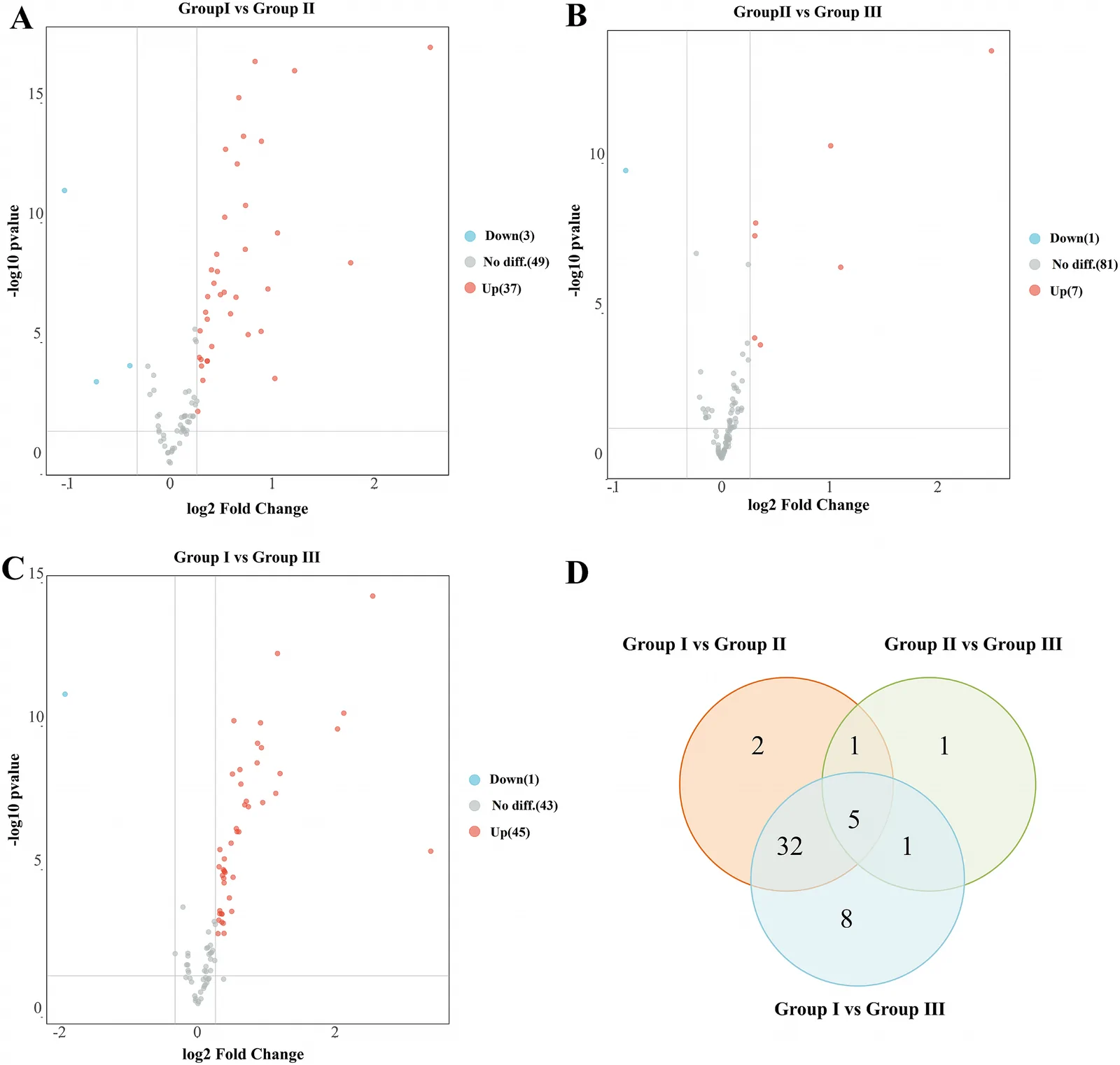
Volcano plots of differential non-volatile components during XYMJ tea processing. (A) Group I vs group II, (B) group II vs group III, (C) group I vs group III. (D) Venn analysis.

### 3.3 Analysis of changes in non-volatile metabolites during the whole processing of XYMJ

#### 3.3.1 Catechins and their derivatives

Numerous studies have reported that catechins and their derivatives are the primary contributors to the bitterness and astringency of tea. As illustrated in Fig. 2C, it was observed that the fixation step (SQ) only increased the content of (−)-Epigallocatechin, which then decreased in subsequent processing steps to a level with no significant difference from that in fresh tea leaves. As processing progressed, only the content of Epiafzelechin reached 1.15 times that in fresh leaves after initial drying, and further increased to 1.23 times after re-drying. The content of (+)-Catechin 3-O-gallate showed a trend of initially increasing and then decreasing as processing advanced. The content of catechins and their derivatives did not undergo substantial changes during the processing of green tea, which is consistent with previous studies (Gui et al., 2023). Notably, the content of epiafzelechin increased significantly (*p* < 0.05) during the initial and re-baking stages. This non-methylated catechin may contribute a distinct sweet aftertaste, which could be one of the reasons for the enhanced sweetness in manufacture of XYMJ green tea (Wei et al., 2022).

#### 3.3.2 Flavonoids and their glycosides

Flavonoids and their glycoside derivatives exhibited two primary change trends during the processing:(1) Significant increases were observed in compounds such as 3,4,2′,4′-tetrahydroxychalcone, cyanidin-3-O-glucoside, cyanidin, kaempferol-3-O-(6”-O-p-coumaroyl)-glucoside, kaempferol-3-O-glucosylgalactoside, kaempferol-3-O-glucosyl-(1 → 2)-rhamnoside, myricetin-3-O-glucoside, and quercetin-3-O-glucofuranoside. (2) 8-Hydroxykaempferol, Kaempferol, Luteolin, Quercetin 3-O-galactoside, and Quercetin 3-O-rutinoside exhibited a trend of initial increase followed by a decrease. Notably, camellianin A was the only compound that showed a consistent decrease throughout processing. Overall, the majority of flavonoids and their glycosylated derivatives increased in content during green tea processing, which aligns with findings reported in previous studies (Gui et al., 2023). As illustrated in Table 1 and the corresponding heatmap (Fig. 2C), a significant rise in most flavonoids and flavonoid glycosides occurred particularly during the fixation stage, which aligns with the increasing trend of these compounds observed during Longjing green tea processing (Zeng et al., 2024). Nevertheless, studies have indicated that heat treatment during processing can lead to a decrease in flavonoid glycoside content (Fang et al., 2019). This may be attributed to the differential rates of synthesis and degradation. The metabolism of glycosides is a dynamic process. In the early processing stages, such as fixation, the breakdown of polymeric compounds or the transformation of other precursors may lead to the generation of kaempferol and kaempferol-3-O-glycoside, resulting in an initial increase in their levels. In contrast, during the drying stage, the enzymatic activity responsible for synthesizing kaempferol-3-O-glycoside from kaempferol declines, leading to a subsequent decrease in kaempferol content. The relevant conversion mechanism requires further research.

#### 3.3.3 Amino acids and their derivatives

Previous study has reported that fixation increases asparagine levels, which contributes to the fresh, brisk quality of tea (Zheng et al., 2025). The content of asparagine increased significantly after fixation, decreased during subsequent rolling and shaping, and rose again during initial drying and re-drying in this study. The D-theanine content showed a decreasing trend during processing of XYMJ green tea, consistent with findings in previous studies. This decline may be attributed to the Maillard reaction between theanine and sugars under thermal treatment, forming aromatic compounds. Alternatively, in this study, the reduction in theanine could be due to its substantial conversion into theanine glucoside (Wang, Hua, et al., 2021). Notably, the content of theanine glucoside increased dramatically during initial and *Re*-drying processing, reaching 7.39 and 13.57 times the level in fresh leaves, respectively. Such an increase is common in green tea processing. For instance, Liu et al. found that the theanine glucoside content in roasted samples was 21.23 times that of unroasted samples (Liu et al., 2023). Nevertheless, the flavor characteristic of theanine glucoside remains insufficient, with limited reports suggesting a potential association between theanine glucoside and the umami presentation in tea (Liu et al., 2023). Consistently, the correlation analysis (Fig. S2) in our study also revealed that theanine glucoside exhibited a positive correlation with umami. Glutamic acid, as key umami compounds, showed a consistent rise from the fixation stage onward (Bellisle, 1999). The variation trends of these amino acids and their derivatives were in line with the electronic tongue results, which recorded a significant increase in the umami intensity of XYMJ green tea during processing.

#### 3.3.4 Organic acids and alkaloids

During the processing of XYMJ green tea, the contents of three organic acids (4-hydroxybenzoic acid, malic acid dimethyl ester, and pheophorbide B) showed a gradually increasing trend. Meanwhile, among the alkaloids, theobromine and sparteine were identified as key differential components in this green tea. However, these two substances exhibited opposite trends during processing: theobromine content progressively decreased, while sparteine content increased significantly. Studies have confirmed that theobromine is a major contributor to the bitterness of tea infusion (Shi et al., 2025; Wen et al., 2024). Theobromine content decreased during processing in the present study, which suggests that tea processing may inhibit the synthesis of theobromine or promote the metabolism of theobromine, thereby contributing to a reduction in the bitterness and astringency of tea. A similar suppressing effect on theobromine has been reported in previous studies using red light during the withering stage (Lin et al., 2022).Therefore, the overall decrease in bitter aftertaste intensity observed for XYMJ green tea in this study may be closely associated with the reduction in theobromine concentration during processing.

#### 3.3.5 Nucleosides and lipids

The adenosine content increased markedly during fixation, reaching 5.83 times that in fresh tea leaves, which was consistent with the findings of (Li et al., 2019). A concurrent rise in adenine and 5′-Methylthioadenosine levels was also observed. While the increase of 5′-Methylthioadenosine in thermally processed green tea aligns with existing literature, the direct influence of 5′-Methylthioadenosine on sensory flavor qualities has not been fully clarified and warrants further study (Ni et al., 2025). The content of gamma-linolenic acid, a common fatty acid in green tea, decreased sharply during the fixation stage and gradually increased in subsequent processing stages.

### 3.4 Analysis of dynamic changes and sweetness contribution of soluble sugars

As sugars constitute the primary contributors to the sweet taste of XYMJ tea, GC–MS-based targeted metabolomics was adopted to characterize the dynamic variations of sugars over the processing of XYMJ green tea. In total, 9 monosaccharides, 4 disaccharides, 1 trisaccharide and 10 sugar derivatives were tentatively identified. The overall variation patterns of these saccharides across successive processing stages are illustrated in Fig. S1. As shown in Fig. 4A, the total soluble sugar content showed a significant increasing trend, reaching its peak (49.77 mg/g) during the re-drying stage, consistent with the variation trend observed during Longjing tea processin (Zeng et al., 2024). Among 14 soluble sugars, monosaccharides were highest in the fresh leaves stage (6.17 mg/g), accounting for 15.59% of the total soluble sugars. Their content dropped sharply to 2.97 mg/g (7.44%) during the fixation process and showed a slight increase in subsequent stages (Fig. 4B). The total disaccharide content demonstrated a clear and continuous increasing trend, rising from 31.49 mg/g in the fresh leaves stage to 42.41 mg/g in the re-drying stage (Fig. 4C). The trisaccharide (raffinose) showed a similar, significantly increasing trend (Fig. 4H). Glucose and d-fructose were the predominant monosaccharides (>1 mg/g), together constituting over 90% of the total monosaccharides, and their contents decreased significantly during fixation (Fig. 4D and E). Disaccharides were the main component of soluble sugars, accounting for 79.62% to 86.64% throughout processing. Sucrose was the primary disaccharide, representing over 90% of the total disaccharides. Its content increased significantly starting from the fixation stage and reached 38.86 mg/g by the re-drying stage, constituting 78.09% of the total soluble sugars (Fig. 4F). Obviously, Sucrose is the most crucial sweet-taste substance in XYMJ green tea.

**Fig. 4:**
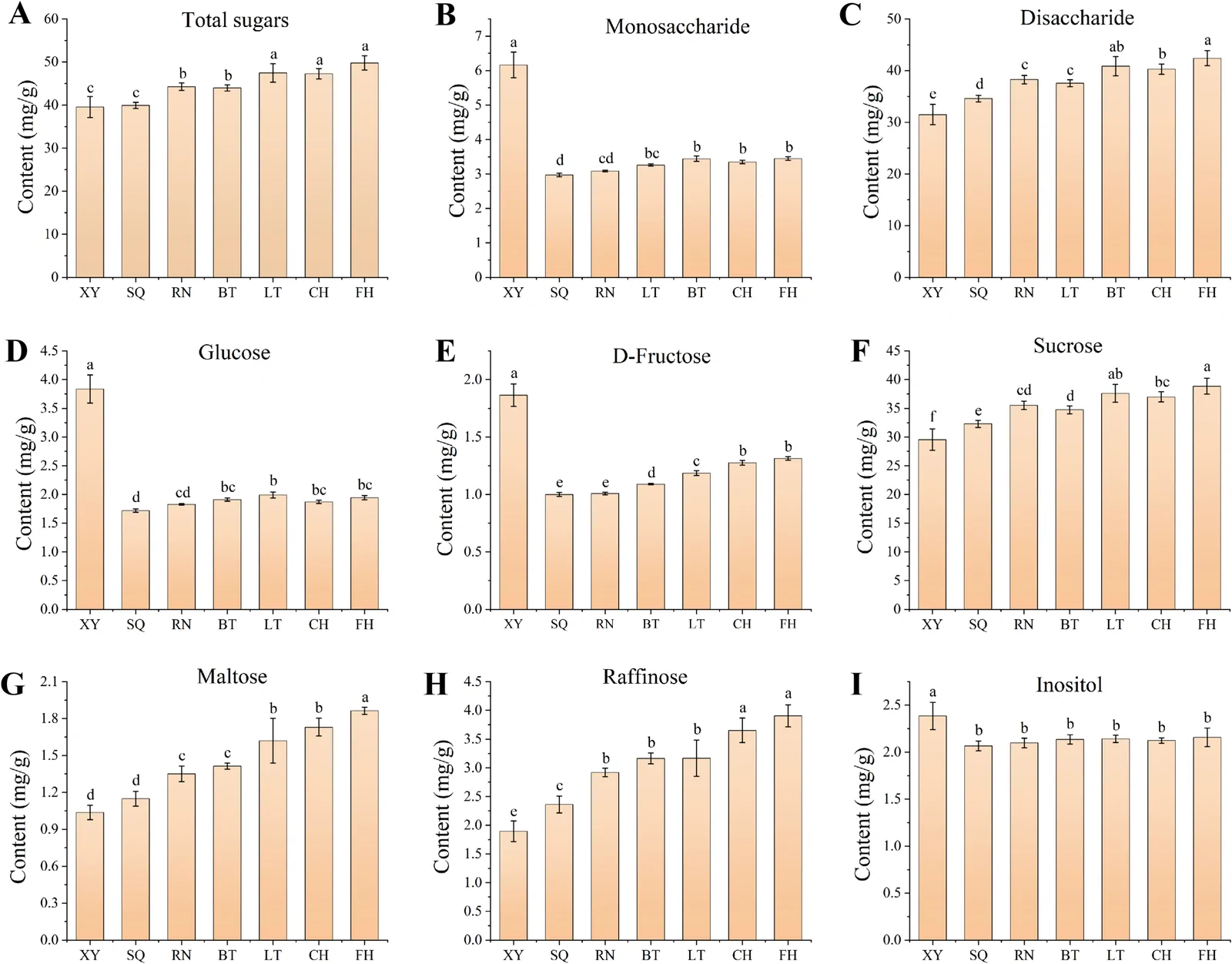
Dynamic metabolic changes of soluble sugars and their derivatives (content >1 mg/g) during the processing of XYMJ green tea. The content of (A) Total soluble sugar, (B) Total monosaccharides, (C) Total disaccharides, (D) Glucose, (E) d-Fructose, (F) Sucrose, (G) Maltose, (H) Raffinose and (I) Inositol. Values marked with different letters (a, b, c…) indicate significant differences between groups (*p* < 0.05). (For interpretation of the references to color in this figure legend, the reader is referred to the web version of this article.)

The transformation of these soluble sugars is closely related to the processing stages. The significant increase in total soluble sugars after fixation is consistent with previous research (Yu et al., 2023). Beginning with the rolling stage, the cellular structure of tea leaves is disrupted. Insoluble sugars such as starch and cell wall polysaccharides are hydrolyzed or thermally degraded under heat and enzymatic actions, converting into soluble disaccharides, which gradually increases the soluble sugar content (Ahmed & Stepp, 2013). Although polysaccharide degradation also produces small amounts of monosaccharides, monosaccharides like glucose and fructose are highly susceptible to participation in the Maillard reaction and caramelization under the high temperatures of fixation and drying, leading to their rapid consumption. Consequently, their consumption rate likely surpasses their production rate, keeping monosaccharide levels relatively low following fixation. The accumulation of sucrose and raffinose may result from the inactivation of their hydrolytic enzymes during heat treatment, which inhibits the hydrolysis pathway while activating the biosynthetic pathway.

As the predominant soluble sugar in XYMJ green tea, sucrose exerts a dominant effect on sweetness development via its dynamic variations during processing. Sucrose and fructose are recognized as the sweetest among conventional saccharides, whereas certain disaccharides including maltose possess higher sweetness than monosaccharides (Moskowitz, 1970). Multiple disaccharides, including sucrose, maltose, and D-cellobiose (all >1 mg/g), displayed continuous accumulation across processing stages, collectively and significantly elevating the sweetness intensity of XYMJ tea. This observation aligns well with the electronic tongue measurements. Although monosaccharides such as d-fructose and glucose showed a significant decreasing trend throughout processing, their relatively low abundance compared to sucrose precluded them from altering the overall rising tendency of sweetness.

### 3.5 Major metabolic pathways during the whole processing of XYMJ

To elucidate the dynamic transformations of non-volatile metabolites during the entire processing of XYMJ tea, a metabolic pathway analysis was performed on differential metabolites with VIP >1 identified during processing. The analysis focused on key compound classes including flavonoids and their glycosides, amino acids, and sugars. As illustrated in Fig. 5, a representative flavonoids conversion pathway involved the glycosylation/acylation of kaempferol, leading to the production of flavonoid glycosides such as kaempferol 3-O-glucosylgalactoside. Regarding amino acids and derivatives, a critical metabolic route consists of the glycosylation of D-theanine to form theanine glucoside, which potentially contributes to enhanced umami taste. The representative metabolic and biosynthetic pathways of sugars include the process in which raffinose, under the action of α-galactosidase, produces D-galactose and sucrose, followed by the hydrolysis of sucrose into glucose and fructose.

**Fig. 5:**
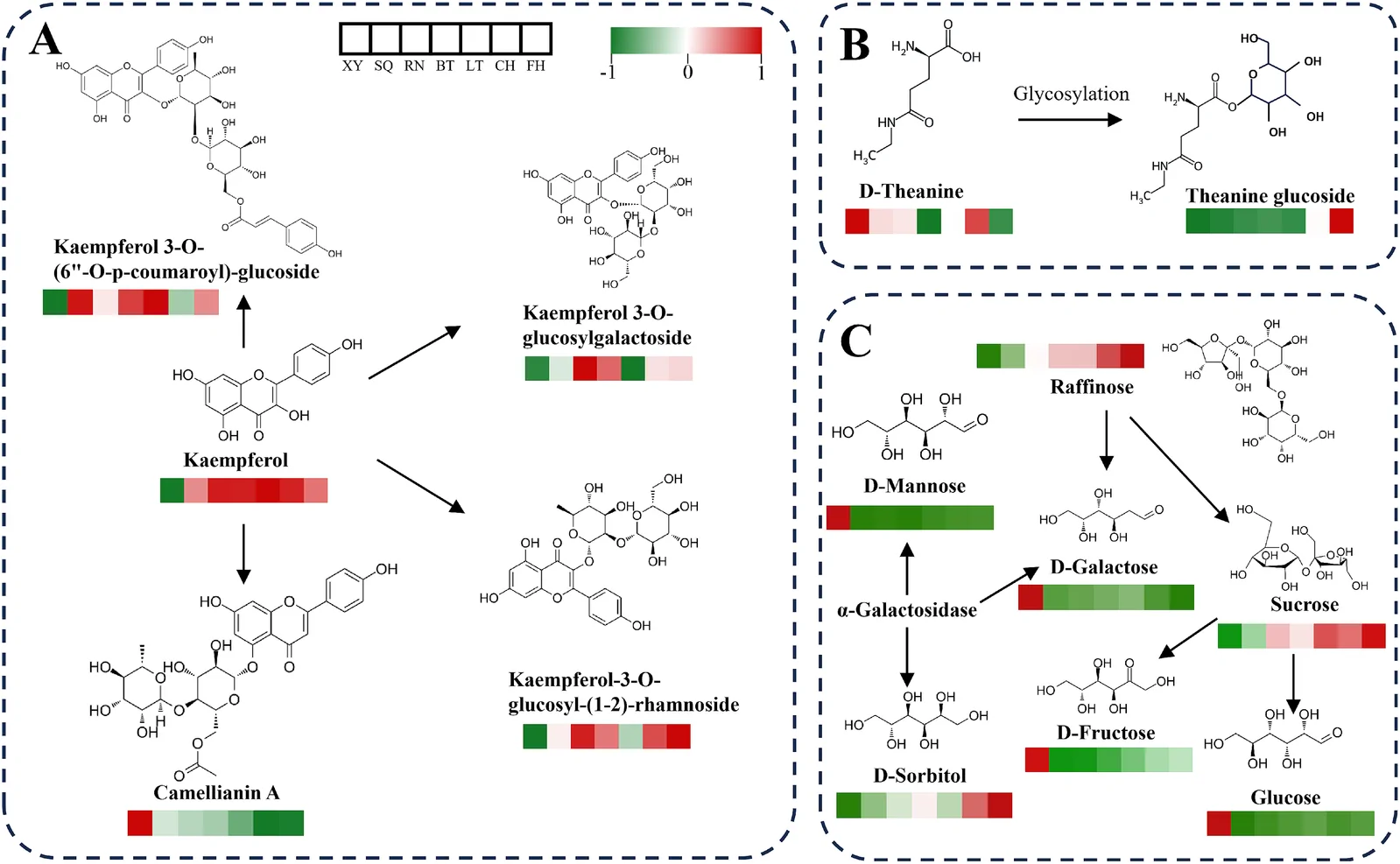
The biosynthesis metabolism of representative (A) flavonoids and their glycosides, (B) amino acids, and (C) sugars during XYMJ tea processing. The relative abundance of metabolites is represented by a color gradient from red (high) to green (low). (For interpretation of the references to color in this figure legend, the reader is referred to the web version of this article.)

## 4 Conclusion

Untargeted metabolomics coupled with multivariate statistical analysis and electronic tongue taste profiling were employed to investigate the taste quality and metabolomic characteristics of XYMJ green tea during processing. A total of 36 differential metabolites were identified throughout the manufacturing stages. Fixation and drying were determined to be the key processing steps governing the formation of XYMJ's taste profile. An exploratory analysis was conducted on the relationship between the dynamic changes of compounds and the evolution of sensory taste. The increase in sweetness may be associated with elevated levels of soluble disaccharides (primarily sucrose), while the enhancement of umami may be related to the accumulation of theanine glycosides and glutamic acid. The reduction in bitter and astringent aftertastes was correlated with decreased contents of theobromine. This study innovatively elucidates the molecular basis underlying the formation of taste quality during the processing of XYMJ green tea. Our findings provide a theoretical foundation for further optimization of its manufacturing protocol. Moreover, newly identified potential quality-modulating compounds, such as theanine glucoside, warrant further investigation into their specific flavor attributes.

## CRediT authorship contribution statement

**Lanlan Peng:** Writing – original draft. **Lizeng Cheng:** Software. **Kang Wei:** Writing – review & editing. **Xinlin Wei:** Funding acquisition, Conceptualization.

## Declaration of competing interest

The authors declare that they have no known competing financial interests or personal relationships that could have appeared to influence the work reported in this paper.

## Data Availability

Data will be made available on request.

## Appendix A Supplementary data

Supplementary material 1 Heatmap analysis of dynamic changes in sugars and their derivatives during the whole processing of XYMJ green tea (Fig. S1). Correlation analysis between key compounds and tastes detected by an electronic tongue (Fig. S2)

Supplementary material 2 The detailed information of the 90 non-volatile metabolites identified in the processing of XYMJ green tea

## References

[bb0005] Ahmed S., Stepp J.R. (2013). Green tea: The plants, processing, manufacturing and production. Tea in Health and Disease Prevention.

[bib146] Ahmed S., Stepp J.R. (2024). Green tea: Plants, artisanal knowledge, processing, manufacturing and production. Tea in Health and Disease Prevention.

[bb0010] Bellisle F. (1999). Glutamate and the UMAMI taste: Sensory, metabolic, nutritional and behavioural considerations. A review of the literature published in the last 10 years. Neuroscience and Biobehavioral Reviews.

[bb0015] Cheng L., Zhu H., Peng L., Li X., Xu L., Yang M., Wei X. (2026). Metabolic conversion and quality contribution of tea-derived *Aspergillus nidulans* and its safety assessment in dark tea manufacture. Journal of Agricultural and Food Chemistry.

[bb0020] Cui J., Zhai X., Guo D., Du W., Gao T., Zhou J., Song C. (2022). Characterization of key odorants in Xinyang Maojian green tea and their changes during the manufacturing process. Journal of Agricultural and Food Chemistry.

[bb0025] Fang Z.T., Song C.J., Xu H.R., Ye J.H. (2019). Dynamic changes in flavonol glycosides during production of green, yellow, white, oolong and black teas from *Camellia sinensis* L. (cv. Fudingdabaicha). International Journal of Food Science and Technology.

[bb0030] Fayaz U., Srivastava S., Dar A.H., Bashir I., Manzoor S., Pandey V.K., Dash K.K. (2024). Recent insights into E-tongue interventions in food processing applications: An updated review. Current Food Science and Technology Reports.

[bb0035] Giordani E., Doumett S., Nin S., Del Bubba M. (2011). Selected primary and secondary metabolites in fresh persimmon (*Diospyros kaki* Thunb.): A review of analytical methods and current knowledge of fruit composition and health benefits. Food Research International.

[bb0040] Gui A., Gao S., Zheng P., Feng Z., Liu P., Ye F., Yin J. (2023). Dynamic changes in non-volatile components during steamed green tea manufacturing based on widely targeted metabolomic analysis. Foods.

[bb0045] Kumar S., Thakur M., Mitra R., Basu S., Anand A. (2022). Sugar metabolism during pre- and post-fertilization events in plants under high temperature stress. Plant Cell Reports.

[bb0050] Li P., Zhu Y., Lu M., Yang C., Xie D., Tan J., Peng Q., Zhang Y., Ni D., Dai W., Lin Z. (2019). Variation patterns in the content of glycosides during green tea manufacturing by a modification-specific metabolomics approach: Enzymatic reaction promoting an increase in the glycosidically bound volatiles at the pan firing stage. Food Chemistry.

[bb0055] Lin J., Liu F., Zhou X., Tu Z., Chen L., Wang Y., Yang Y., Wu X., Lv H., Zhu H., Ye Y. (2022). Effect of red light on the composition of metabolites in tea leaves during the withering process using untargeted metabolomics. Journal of the Science of Food and Agriculture.

[bb0060] Liu F., Tu Z., Chen L., Lin J., Zhu H., Ye Y. (2023). Analysis of metabolites in green tea during the roasting process using non-targeted metabolomics. Journal of the Science of Food and Agriculture.

[bb0065] Moskowitz H.R. (1970). Ratio scales of sugar sweetness. Attention, Perception & Psychophysics.

[bb0070] Ni Z., Pan H., Chen Y., Chen J., Wu W., Chen W., Zhou J. (2025). Metabolomics and sensomics reveal non-volatile flavor dynamics in summer–autumn steamed green tea flakes processing. npj Science of Food.

[bb0075] Pan S.Y., Nie Q., Tai H.C., Song X.L., Tong Y.F., Zhang L.J.F., Liang C. (2022). Tea and tea drinking: China’s outstanding contributions to the mankind. Chinese Medicine.

[bb0080] Shan X., Deng Y., Niu L., Chen L., Zhang S., Jiang Y., Yuan H., Wang Y., Li J. (2024). The influence of fixation temperature on Longjing tea taste profile and the underlying non-volatile metabolites changes unraveled by combined analyses of metabolomics and E-tongue. LWT.

[bb0085] Shi X., Xiao Y., Zhao N., Yao L., Li H., Huang J., Song Z., Wang X., Wang X. (2025). Bitter and astringent characteristics of alkalized cocoa powder: Analysis using UPLC-Q-TOF-MS/MS and molecular simulation. Food Chemistry.

[bb0090] Steiner D., Meyer A., Immohr L.I., Pein-Hackelbusch M. (2024). Critical view on the qualification of electronic tongues regarding their performance in the development of peroral drug formulations with bitter ingredients. Pharmaceutics.

[bb0095] Wang H., Cao X., Yuan Z., Guo G. (2021). Untargeted metabolomics coupled with chemometrics approach for Xinyang Maojian green tea with cultivar, elevation and processing variations. Food Chemistry.

[bb0100] Wang H., Hua J., Yu Q., Li J., Wang J., Deng Y., Yuan H., Jiang Y. (2021). Widely targeted metabolomic analysis reveals dynamic changes in non-volatile and volatile metabolites during green tea processing. Food Chemistry.

[bb0105] Wei Y., Yin X., Wu H., Zhao M., Huang J., Zhang J., Li T., Ning J. (2022). Improving the flavor of summer green tea (*Camellia sinensis* L.) using the yellowing process. Food Chemistry.

[bb0110] Wen S., Bai S., An R., Peng Z., Chen H., Jiang R., Liu Z. (2024). Key metabolites influencing astringency and bitterness in Yinghong 9 large-leaf dark tea before and after pile-fermentation. Journal of Agricultural and Food Chemistry.

[bb0115] Wu H., Ren Q., Tang X., Zhang H., Zhao C., Yang J., Zheng J. (2020). Research progress of tea functional components in the prevention and treatment of lung cancer. Food and Health.

[bb0120] Yin P., Kong Y.S., Liu P.P., Jiang C.L., Sun M.F., Guo G.Y., Liu Z.H. (2022). Temporal variation of the non-volatile compounds and key odorants in Xinyang Maojian green teas during the spring and autumn seasons. Agronomy.

[bb0125] Yin P., Wang J.J., Kong Y.S., Zhu Y., Zhang J.W., Liu H., Liu Z.H. (2022). Dynamic changes of volatile compounds during the Xinyang Maojian green tea manufacturing at an industrial scale. Foods.

[bb0130] Yu Y., Zhu X., Ouyang W., Chen M., Jiang Y., Wang J., Hua J., Yuan H. (2023). Effects of electromagnetic roller-hot-air–steam triple-coupled fixation on reducing the bitterness and astringency and improving the flavor quality of green tea. Food Chemistry: X.

[bb0135] Zeng L., Fu Y.Q., Gao Y., Wang F., Liang S., Yin J.F., Xu Y.Q. (2024). Dynamic changes of key metabolites in Longjing green tea during processing revealed by widely targeted metabolomic profiling and sensory experiments. Food Chemistry.

[bb0140] Zhang J., Feng W., Xiong Z., Dong S., Sheng C., Wu Y., Ning J. (2024). Investigation of the effect of over-fired drying on the taste and aroma of Lu’an Guapian tea using metabolomics and sensory histology techniques. Food Chemistry.

[bb0145] Zheng Y., Zhang Y., Zou Y., Shi Y., Zhang J., Deng H., Li X. (2025). Chemical profiling and sensory analysis reveal quality differentiation in Baimudan white tea processed from three major Fujian tea cultivars. Horticulturae.

