# Supplementary material for: Integrated metabolomics and electronic tongue analysis reveal dynamic changes in taste characteristics and non-volatile metabolites during Xinyang Maojian green tea processing
Source: Food Chem X. 2026 Oct 14;40:104601. doi: 10.1016/j.fochx.2026.104601 (PMC13691147; doi:10.1016/j.fochx.2026.104601)
Supplement: Supplementary file 1 [file mmc1.docx]

***Integrated Metabolomics and Electronic Tongue Analysis Reveal Dynamic Changes in Taste Characteristics and Non-volatile Metabolites during Xinyang Maojian Green Tea Processing***

*Lanlan Peng ^a^, Lizeng Cheng ^a^, Yang Wei ^a^, Kang Wei ^a*^, Xinlin Wei ^a*^*

*^a^ School of Agriculture and Biology, Shanghai Jiao Tong University, 800 Dongchuan Road, Shanghai 200240, PR China*

*^#^* These authors contributed equally to this work.

*^*^*Corresponding author.

Tel.: + 86-21-34208533

Address: No. 800 Dongchuan Road, Minhang District, Shanghai 201100, China

E-mail: foodlab2010@163.com

*^*^*Corresponding author.

Tel.: +86-21-34208533

Address: No. 800 Dongchuan Road, Minhang District, Shanghai 201100, China

E-mail: wk5516828@sjtu.edu.cn

**Fig. S1.** The heatmap analysis of the 25 differential sugars throughout the processing stages of XYMJ, and the relative abundance of metabolites is shown by a color gradient from red (high) to green (low)

**Fig. S2** Correlation analysis between key compounds and tastes detected by an electronic tongue. **represents significant differences (p < 0.01). *** represents significant differences (p < 0.001).
